# A new scoring model for the prediction of mortality in patients with acute kidney injury

**DOI:** 10.1038/s41598-017-08440-w

**Published:** 2017-08-11

**Authors:** Min Luo, Yuan Yang, Jun Xu, Wei Cheng, Xu-Wei Li, Mi-Mi Tang, Hong Liu, Fu-You Liu, Shao-Bin Duan

**Affiliations:** Department of Nephrology, The Second Xiangya Hospital, Central South University, Changsha, Hunan 410011 China

## Abstract

Currently, little information is available to stratify the risks and predict acute kidney injury (AKI)-associated death. In this present cross-sectional study, a novel scoring model was established to predict the probability of death within 90 days in patients with AKI diagnosis. For establishment of predictive scoring model, clinical data of 1169 hospitalized patients with AKI were retrospectively collected, and 731 patients of them as the first group were analyzed by the method of multivariate logistic regression analysis to create a scoring model and further predict patient death. Then 438 patients of them as the second group were used for validating this prediction model according to the established scoring method. Our results showed that Patient’s age, AKI types, respiratory failure, central nervous system failure, hypotension, and acute tubular necrosis-individual severity index (ATN-ISI) score are independent risk factors for predicting the death of AKI patients in the created scoring model. Moreover, our scoring model could accurately predict cumulative AKI and mortality rate in the second group. In conclusion, this study identified the risk factors of 90-day mortality for hospitalized AKI patients and established a scoring model for predicting 90-day prognosis, which could help to interfere in advance for improving the quality of life and reduce mortality rate of AKI patients.

## Introduction

Acute kidney injury (AKI) is a common complication among hospitalized patients, and is an important cause for in-hospital death. In recent years, the incidence of AKI continues increasing at an annual growth rate of 11%^1^. In the past 25 years, this incidence has increased by at least 20 times^2^. AKI has a death rate as high as 20%, which may reach up to 50% in the intensive care unit (ICU)^3, 4^. Each year, around two million people die of AKI worldwide^5^. AKI is not only a medical problem, but also has become a major public health concern.

Referring to different diagnostic criteria of AKI, currently there are 35 descriptions recorded in literature worldwide^6, 7^. The acute kidney injury network (AKIN) criteria are further formulated according to the RIFLE criterion (risk, injury, failure, loss, end stage renal disease)^8^. Evidently, RIFLE and AKIN criteria have made tremendous contributions for the diagnosis and treatment of AKI^9^. However, due to various restrictions, AKI was redefined on the basis of RIFLE and AKIN criteria by the Kidney Disease Improving Global Organization (KDIGO)^8^. In March 2012 the “KDIGO acute kidney injury clinical practice guidelines” was released^10^. Studies had identified that the outcome prediction performance of KDIGO classification is superior to that of AKIN or RIFLE classification in critically ill patients^8, 11^. The patients were diagnosed and classified again according to the latest KDIGO clinical practice guidelines in our research.

The representative AKI prognosis scoring systems included Acute Physiology and Chronic Health Evaluation (APACHE II), Simplified Acute Physiology Score (SAPS II), Mortality Probability Models (MPM II), Acute tubular necrosis-individual severity degree index (ATN-ISI), Sequential Organ Failure Assessment (SOFA) and Stuivenberg hospital acute renal failure scores (SHARF) can predict outcomes in patients with AKI. Most study index of the above scoring systems were derived from ICU and were more suitable for the critically ill patients with multiple organ failures in ICU^12–14^. Therefore, the aims of present study were to further evaluate the risk factors of death for hospitalized AKI patients and establish a new scoring model for predicting 90-day mortality rate after AKI diagnosis according to KDIGO criteria.

## Methods

### Study design and patient population

**Figure 1:**
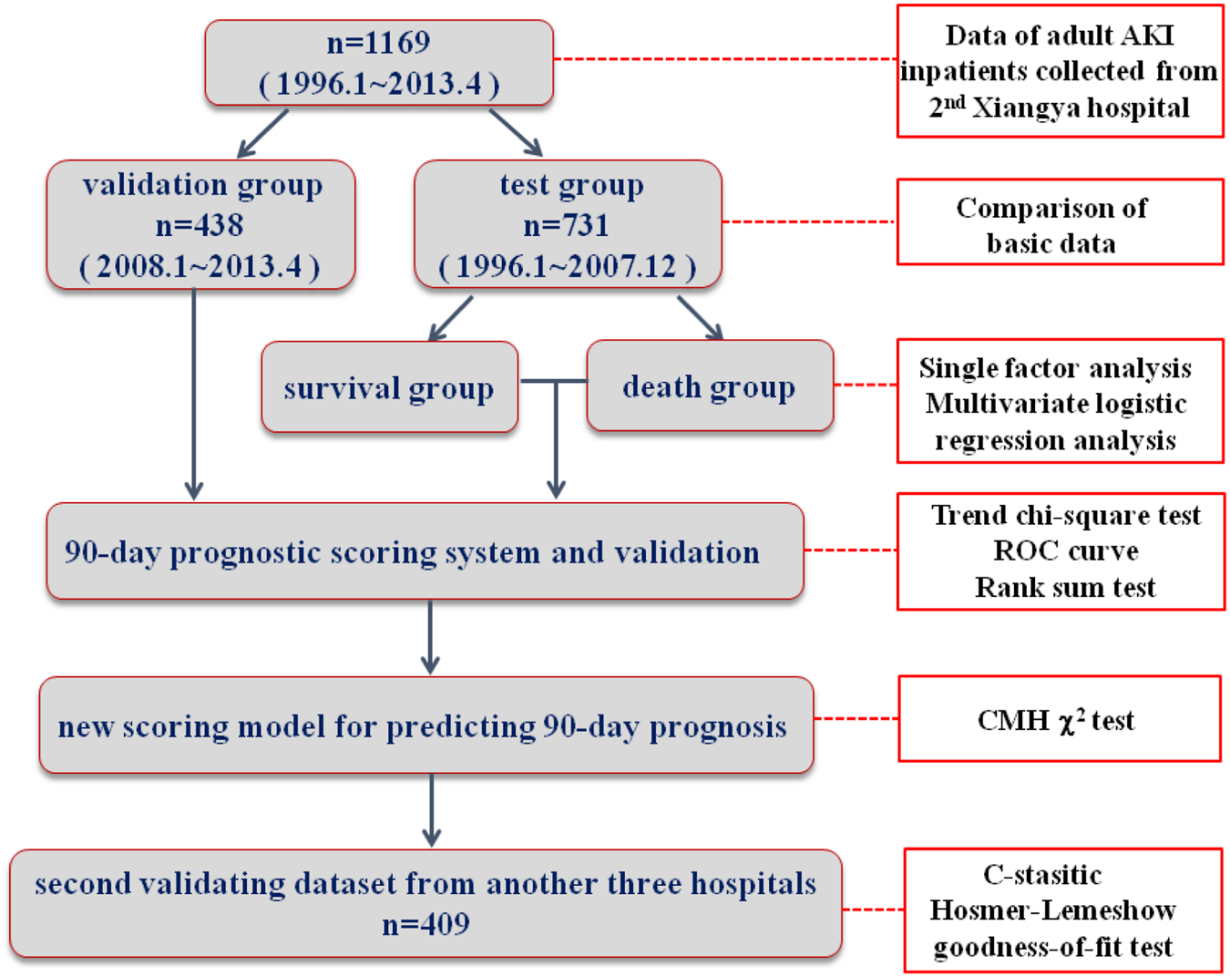
Flow chart of study population selection and research process.

1169 cases of AKI inpatients admitted in the Second Xiangya Hospital of Central South University were selected from January 1996 to April 2013, and all conformed to the KDIGO criteria. A total of 731 patients were treated as the test group, including 454 males and 277 females with an average age of 48.49. And 438 cases were treated as the validation group, including 266 males and 172 females, with an average age of 52.66. Patients with uncompleted medical history or lack of basic information, with stages 5 chronic kidney disease (CKD) and those receiving maintenance dialysis or renal transplantation were excluded. For patients with multiple hospitalizations, we only included only the first hospitalization in the analysis set^15^. The medical ethics community of the Second Xiangya Hospital approved the study protocol and waived patient consent. AKI types included hospital-acquired AKI and community-acquired AKI^16^. Flow chart of study population selection and research process was shown in Fig. 1. In addition, we established another validating dataset containing 409 AKI patients hospitalized in another three hospitals (Xiangya Hospital, Third Xiangya Hospital of Central South University and the First Affiliated Hospital of Hunan University of Chinese Medicine) from January 2015 to June 2015 and further validated the predictive performance, stability and repeatability of new score, basic data was shown in Supplementary Table S1.

### Study parameters, endpoint and outcomes

The patients’ data, including age, gender, AKI types, causes of AKI, urine volume, past medical history (CKD, diabetes, hypertension, etc.), mechanical ventilation, sepsis, shock, organ failure, laboratory indexes on admission (hemoglobin, serum albumin, blood urea nitrogen (BUN) peak value, Scr peak value, serum potassium ions (K^+^) peak value, etc.), ATN-ISI, hospital stay, renal replacement therapy (RRT), clinical outcome and other clinical data were analyzed. The observation starting point was community-acquired AKI 24 hours after admission to hospital or hospital-acquired AKI diagnosis after 24 hours. The observation endpoint was patients’ death or 90 days after AKI diagnosis. The survival state (survive or death) during 90 days were calculated as the endpoint event, ruling out accident harm to death *et al*.

### Definitions

AKI (based on the KDIGO classification); CKD (based on abnormalities of kidney function or structure, and present for >3 months; Proteinuria (defined as exceed 150 mg/24 h); Baseline Scr was the first value measured during hospitalization or the value within 3 months at most 1 year when baseline Scr was unknown within 1 week during hospitalization, the estimated GFR was calculated according to MDRD equation^17, 18^.

Oliguria (defined as urine volume <400 ml/24 h); Anuria (defined as urine volume <100 ml/24 h); Shock (defined as hypotension with systolic arterial blood pressure of 90 mm Hg despite adequate fluid resuscitation); Heart failure (based on Framingham criteria and defined as New York Heart Association functional class IV); Respiratory failure (need for mechanical ventilation); Gastrointestinal failure (stress ulcers requiring transfusion, acalculus cholecystitis); Central nervous system failure (Progressive coma); Hepatic failure (clinical jaundice with bilirubin. 8–l0 mg/dL).

Cardio-renal syndromes was defined by Acute Dialysis Quality Initiative consensus group; Hepatorenal syndrome: was defined according to the European Association of Liver Study criteria; sepsis (according to the American College of Chest Physicians – Society of Critical Care Medicine consensus definition); Organic kidney disease: renal parenchymal diseases including glomerular disease and renal microvascular disease, renal vascular disease, acute interstitial nephritis, intratubular obstruction except for ATN. Acute tubular necrosis was arisen as a consequence of septic, toxic, or ischemic insult. Post-renal obstruction: bladder outlet obstruction, tumors, renal calculi, papillary necrosis, retroperitoneal fibrosis^19–21^.

### Statistical analysis

The collected data were employed to establish a qualified database and were statistically analyzed using SPSS 19.0 and SAS 9.3. The data of normal distribution were presented using mean ± standard deviation. The data without abnormal distribution were shown using the median and inter-quartile range (IQR) and statistically analyzed after logarithmic transformation. The enumeration data were shown using the rate and chi-square test. The mean comparison between the two groups was conducted using the Student’s t-test. The mortality related independent risk factors were analyzed using the multivariate logistic regression analysis. The trend test was conducted using chi-square trend test. The rank sum test was used to determine the difference between groups. CMH χ^2^ test (Cochran mantel haeszel statistics) was used to verify the predictability of forewarning model. In addition, the Hosmer-Lemeshow goodness-of-fit test was used for calibration when evaluating the number of observed and predicted deaths in AKI patients for the entire range of death probabilities. Discrimination was assessed using the area under a receiver operating characteristic curve (AUROC), which was compared with a nonparametric approach. The AUROC analysis calculated cutoff values, sensitivity, specificity, and positive predictive value. Finally, Youden index was defined as Sensitivity + Specificity −1, and the cutoff point was chosen by the best Youden index^22^. A p value of less than 0.05 was considered to be statistically significant.

## Results

### Baseline characteristics

A total of 1169 patients were included, including 720 males and 449 females. The age ranged from 15 to 93, and the average age was 50.05. There were 731 cases in the test group, including 454 males and 277 females. The age ranged from 15 to 93 with an average age of 48.49. There were 438 patients in the validation group, including 266 males and 172 females. The age ranged from 15 to 90 with an average age of 52.66. There was no significant difference in various indexes between the two groups (P > 0.05). The results were shown in Table 1. We also found that the length of hospital stay had no correlation with death rate of hospitalized AKI patients (Table 2).

**Table 1:** Comparison of basic data between the test group and validation group.

parameter	test group (n = 731)	validation group (n = 438)	P value
Age (years, %)			0.861
15~39	170 (23.3%)	108 (24.7%)	
40~64	363 (49.7%)	213 (48.6%)	
≥65	198 (27.1%)	117 (26.7%)	
Gender (male, %)	454 (62.1%)	266 (60.7%)	0.640
Baseline Scr (μmol/L)	102.3 ± 30.4	100.4 ± 31.8	0.302
Baseline eGFR (ml/min/1.73 m²)	73.9 ± 20.9	72.4 ± 18.4	0.205
AKI types			0.743
CA-AKI	660 (90.3%)	398 (90.9%)	
HA- AKI	71 (9.7%)	40 (9.1%)	
Causes of AKI			0.592
hypovolemia	112 (15.3%)	64 (14.6%)	
cardiorenal syndrome	44 (6%)	32 (7.3%)	
hepatorenal syndrome	19 (2.6%)	15 (3.5%)	
sepsis	29 (4.0%)	27 (6.2%)	
organic kidney disease	154 (21.0%)	82 (18.7%)	
acute tubular necrosis	168 (23.0%)	93 (21.2%)	
post-renal obstruction	138 (18.9%)	81 (18.5%)	
multi-factorial	67 (9.2%)	44 (10.0%)	
Proteinuria	335 (45.8%)	197 (45%)	0.777
Hematuresis	440 (60.2%)	278 (63.5%)	0.265
Oliguria/anuria (%)	457 (62.5%)	249 (56.8%)	0.055
CKD(%)(eGFR < 60 ml/min/1.73 m²)	141 (19.3%)	88 (20.1%)	0.738
Diabetes mellitus (%)	38 (5.2%)	23 (5.3%)	0.969
Hypertension (%)	96 (13.1%)	61 (13.9%)	0.700
Mechanical ventilation (%)	84 (11.5%)	40 (9.1%)	0.205
Hypotension (%)	122 (16.7%)	61 (13.9%)	0.208
Shock (%)	31 (4.2%)	24 (5.5%)	0.333
Organ failure (%)			
heart failure	74 (10.1%)	42 (9.6%)	0.768
hepatic failure	37 (5.1%)	12 (2.7%)	0.055
respiratory failure	89 (12.2%)	42 (9.6%)	0.175
gastrointestinal failure	10 (1.4%)	4 (0.9%)	0.489
CNS failure	26 (3.6%)	19 (4.3%)	0.502
Hemoglobin < 90 g/L (%)	175 (24.0%)	107 (24.4%)	0.850
Hypoalbuminemia (%)	306 (41.9%)	183 (41.8%)	0.979
BUN peak value (mmol/L)	27.0 ± 12.2	26.7 ± 12.7	0.712
Scr peak value (μmol/L)	686.9 ± 373.7	683.3 ± 402.1	0.875
Serum K^+^ peak value (mmol/L)	4.9 ± 0.9	4.8 ± 1.1	0.184
hospital stay (days)	15.8 ± 13.7	16.3 ± 16.8	0.656
Renal replacement therapy (%)	410 (56.1%)	258 (58.9%)	0.346

Abbreviation: Scr: serum creatinine; eGFR: estimated glomerular filtration rate; CA-AKI: Community-acquired AKI; HA-AKI: Hospital-acquired AKI; CKD: chronic kidney disease; BUN: Blood urea nitrogen; CNS: central nervous system.

**Table 2:** Relationship between length of hospital stay and 90-day mortality rate in patients with acute kidney injury.

Hospital stay (day)	Death(n)	Survival(n)	Total(n)	Death rate (%)	Chi-square	p
2–10	68	417	485	14.0	0.899	0.638
11–20	48	356	404	11.9		
>21	36	244	280	12.9		
total	152	1017	1169	13.0		

### Comparison of prognostic parameters within 90 days after AKI diagnosis between survival group and death group in patients with acute kidney injury

As shown in Table 3, there was no statistical difference between the two groups in gender, baseline Scr, baseline eGFR, KDIGO staging, proteinuria, hematuresis, oliguria or anuria, CKD, diabetes mellitus, hypertension, sepsis, hepatic failure, Hb < 90 g/L, hypoalbuminemia, hospital stay, Scr peak value and replacement therapy between two groups (P > 0.05). Age, AKI types, causes of AKI, mechanical ventilation, hypotension, shock, heart failure, respiratory failure, digestive failure, central nervous system failure, BUN peak value, K^+^ peak value and ATN-ISI score had significantly statistical difference (P < 0.05).

**Table 3:** Single factor analysis of prognosis within 90 days between the survival group and death group in AKI patients.

parameter	Survival group (n = 630)	Death group (n = 101)	*P* value
Age (years, %)			0.001
15~39	157 (24.9%)	13 (12.9%)	
40~64	317 (50.3%)	46 (45.5%)	
≥65	156 (24.8%)	42 (41.6%)	
Gender (male, %)	388 (61.6%)	66 (65.3%)	0.470
Baseline Scr (μmol/L)	103.1 ± 30.8	97.7 ± 27.8	0.096
Baseline eGFR (ml/min/1.73 m²)	73.6 ± 21.0	76.2 ± 20.4	0.240
AKI types			0.000
CA- AKI	586 (93.0%)	74 (73.3%)	
HA- AKI	44 (7.0%)	27 (26.7%)	
Causes of AKI			0.000
hypovolemia	81 (12.9%)	31 (30.7%)	
cardiorenal syndrome	36 (5.7%)	8 (8.0%)	
hepatorenal syndrome	14 (2.2%)	5 (5.0%)	
sepsis	23 (3.7%)	6 (5.9%)	
organic kidney disease	138 (21.9%)	16 (15.8%)	
acute tubular necrosis	150 (23.8%)	18 (17.8%)	
post-renal obstruction	127 (20.2%)	11 (10.9%)	
multi-factorial	61 (9.6%)	6 (5.9%)	
KDIGO staging			0.128
1	23 (3.7%)	4 (4.0%)	
2	56 (8.9%)	3 (3.0%)	
3	551 (87.4%)	94 (93.0%)	
Proteinuria	290 (46.0%)	45 (44.6%)	0.782
Hematuresis	381 (60.5%)	59 (58.4%)	0.695
Oliguria/anuria (%)	396 (62.9%)	61 (60.4%)	0.635
CKD(%)(eGFR < 60 ml/min/1.73 m²)	115 (18.3%)	26 (25.7%)	0.077
Diabetes mellitus (%)	30 (4.8%)	8 (7.9%)	0.184
Hypertension (%)	83 (13.2%)	13 (12.9%)	0.933
Mechanical ventilation (%)	49 (7.8%)	35 (34.7%)	0.000
Hypotension (%)	76 (12.1%)	46 (45.5%)	0.000
Shock (%)	18 (2.9%)	13 (12.9%)	0.000
Organ failure (%)			
heart failure	43 (6.8%)	31 (30.7%)	0.000
hepatic failure	28 (4.4%)	9 (8.9%)	0.057
respiratory failure	40 (6.3%)	49 (48.5%)	0.000
gastrointestinal failure	3 (0.5%)	7 (6.9%)	0.000
CNS failure	10 (1.6%)	16 (15.8%)	0.000
Hemoglobin <90 g/L (%)	145 (23.1%)	30 (29.7%)	0.149
Hypoalbuminemia (%)	267 (42.4%)	39 (38.6%)	0.476
BUN peak value (mmol/L)	26.4 ± 11.9	30.9 ± 13.8	0.002
Scr peak value (μmol/L)	684.5 ± 384.6	702.3 ± 298.5	0.593
Serun K^+^ peak value (mmol/L)	4.8 ± 0.9	5.2 ± 0.9	0.000
ATN-ISI score	0.14 ± 0.15	0.37 ± 0.25	0.000
Hospital stay (days)	15.8 ± 13.4	15.9 ± 15.5	0.994
Renal replacement therapy (%)	348 (55.2%)	62 (61.4%)	0.248

Abbreviation: AKI: acute kidney injury; Scr: serum creatinine; eGFR: basic estimated glomerular filtration rate; CA-AKI: Community-acquired AKI; HA-AKI: Hospital-acquired AKI; CKD: chronic kidney disease; BUN: Blood urea nitrogen; CNS: central nervous system; ATN-ISI: acute tubular necrosis-individual severity index.

### All-cause mortality rate between the test group and validation group in patients with acute kidney injury

The all-cause mortality in the test group and validation group were counted in 90 days. In the test group, 101 cases occurred to the all-cause death within 90 days, showing a mortality rate of 13.8%; in the validation group, 51 cases occurred in the all-cause death within 90 days manifesting a mortality rate of 11.6%. Our results indicated that there is no significant difference between all-cause mortality of the two group (p > 0.05).

### Establishment of the death independent risk factors in the test group within 90 days after AKI diagnosis in patients with acute kidney injury

The basic data of 90-day prognosis in the test group were compared between the survival group and death group. The parameters including age, AKI types, causes of AKI, mechanical ventilation, hypotension, shock, heart failure, respiratory failure, gastrointestinal failure, central nervous system failure, BUN peak value, K^+^ peak value and ATN-ISI score between the two groups showed statistical significance (p < 0.05). The further multivariate logistic regression analysis indicated that age, AKI types, respiratory failure, central nervous system failure, hypotension, ATN-ISI score were the independent risk factors of death (shown in Table 4). The corresponding integrals of various odds ratio (OR) values were endowed according to the principle of round.

**Table 4:** Multivariate logistic regression analysis of prognosis within 90 days in patients with acute kidney injury in the test group.

Factors	β	SE	Wals	P	OR	95%CI
Age						
15~39			9.828	0.007		
40~64	0.750	0.398	3.547	0.045	2.117	0.970~4.620
≥65	1.281	0.417	9.435	0.002	3.599	1.590~8149
AKI types	0.777	0.350	4.946	0.026	2.176	1.097~4.317
Respiratory failure	1.756	0.363	23.452	0.000	5.790	2.844~11.785
CNS failure	1.136	0.546	4.334	0.037	3.114	1.069~9.072
Hypotension	1.025	0.370	7.688	0.006	2.787	1.350~5.750
ATN-ISI score	0.885	0.411	4.635	0.031	2.422	1.083~5.421

Abbreviation: AKI: acute kidney injury; CNS: central nervous system; ATN-ISI: acute tubular necrosis-individual severity index.

### Establishment, trend and fitting degree tests of prognostic scoring system within 90 days after AKI diagnosis in patients with acute kidney injury

Each patient’s score was calculated. As shown in Table 5, the formula was as follow: score of 90-day prognosis = the points of age + the points of AKI types + the points of respiratory failure + the points of central nervous system failure + the points of hypotension + the points of ATN-ISI score. The scoring criteria were as follow: 0 point for age 15~39, 2 points for age 40~64, 4 points for age greater than 65; 0 point for community-acquired AKI and 2 points for hospital-acquired AKI; 6 points for having respiratory failure, and 0 point for not having respiratory failure; 3 points for having central nervous system failure, and 0 point for not having central nervous system failure; 3 points for having hypotension, and 0 point for not having hypotension; 0 point for ATN-ISI score < 0.4 and 2 points for ≥0.4. The score sum of each patient and the mortality rate of each score were respectively calculated.

**Table 5:** Prognostic score of 90-day mortality in hospitalized patients with acute kidney injury.

factors	Classification	points
Age		
	15~39	0
	40~64	2
	≥65	4
AKI types		
	Community-Acquired	0
	Hospital- acquired	2
Respiratory failure		
	no	0
	yes	6
Central nervous system failure		
	no	0
	yes	3
Hypotension		
	no	0
	yes	3
ATN-ISI score		
	<0.4	0
	≥0.4	2
		Maximum 20 points

**Figure 2:**
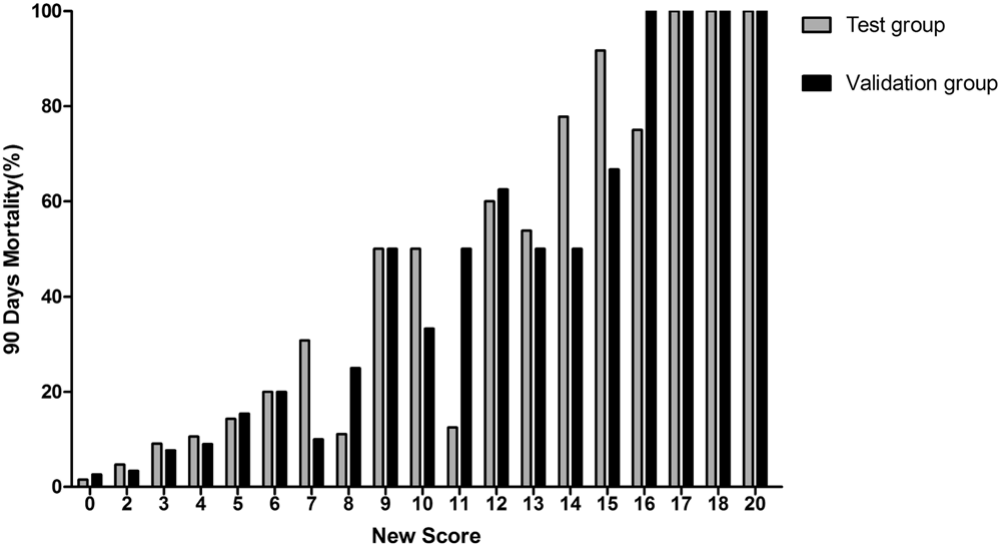
Corresponding 90-day morality for each score between test group and validation group.

**Figure 3:**
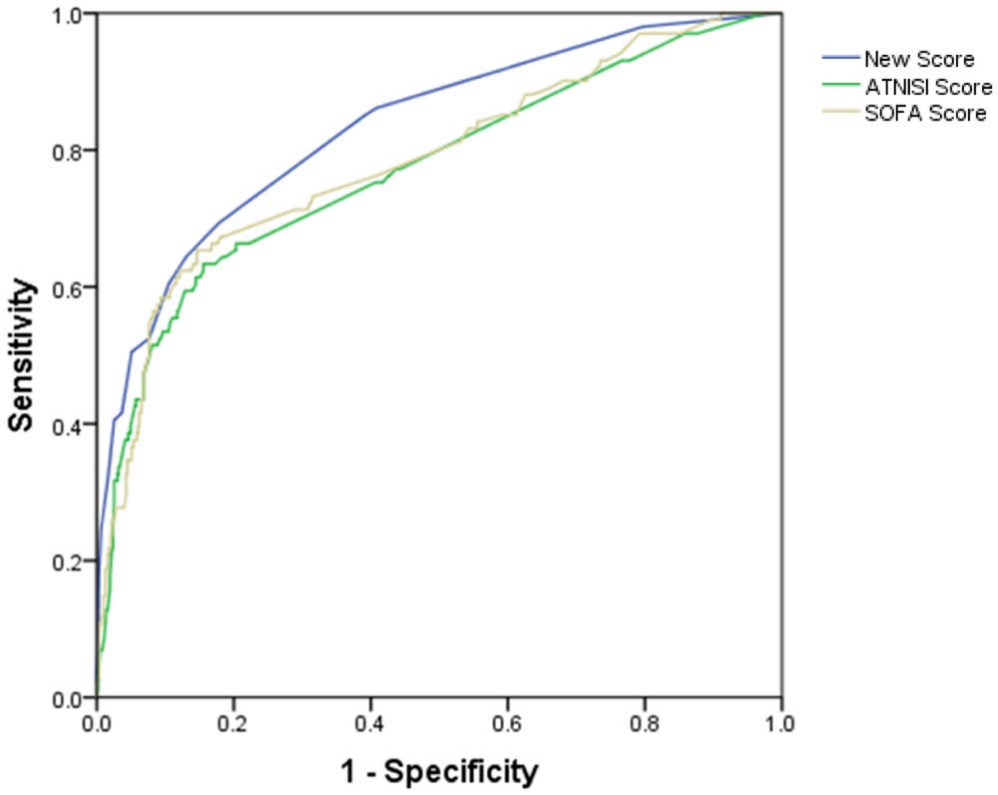
Comparison of areas under the receiver operating characteristic curve among new scores, SOFA and ATN-ISI in test group.

**Figure 4:**
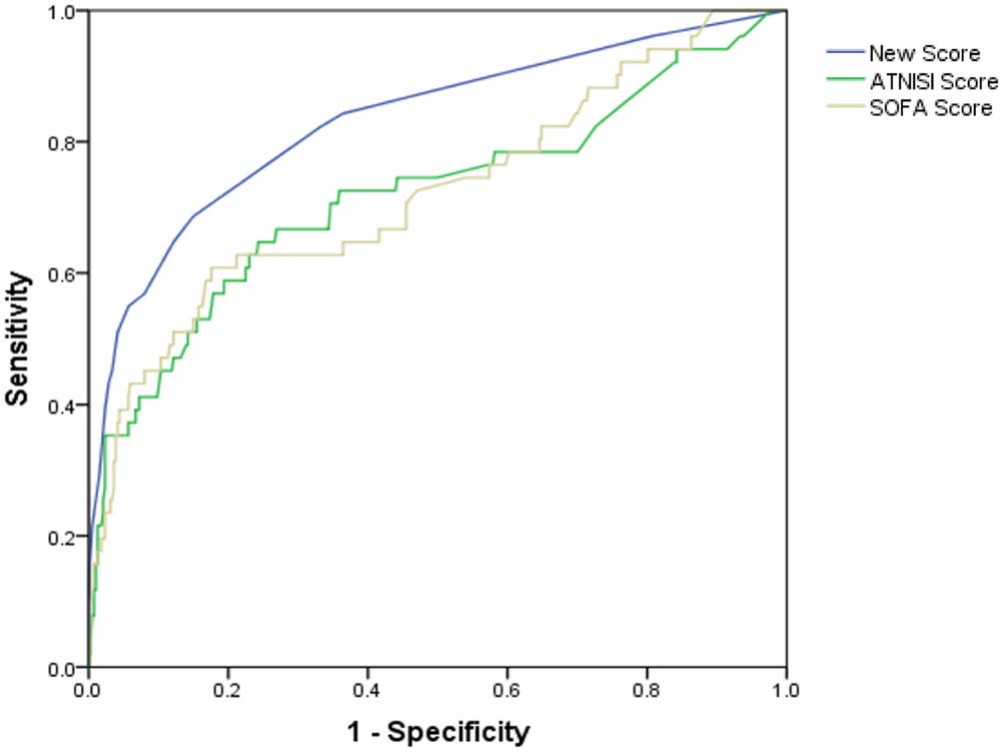
Comparison of areas under the receiver operating characteristic curve among new scores, SOFA and ATN-ISI in validation group.

With the increase of the total score, the mortality rate in the test group was increased. The trend was shown in Fig. 2. The result showed that the mortality rates had statistical significance (P < 0.01). It was predicted that the area under ROC curve of 90-day mortality rate was 0.833 (95% CI: 0.788~0.879), P < 0.001, showing the predictability of the scoring system was reliable. The chi-square trend test was used in the validation group and the result showed that the mortality rates had statistical significance (P < 0.01). It was predicted that the area under ROC curve of 90-day mortality rate was 0.832 (95% CI: 0.764~0.901), P < 0.01. The mortality rates compared between the test group and validation group using rank sum test showed no statistical significance (P = 0.907), and the fitting degree was good (Table 7, Figs 3 and 4).

### Establishment and preliminary application of 90-day prognosis forewarning model

**Figure 5:**
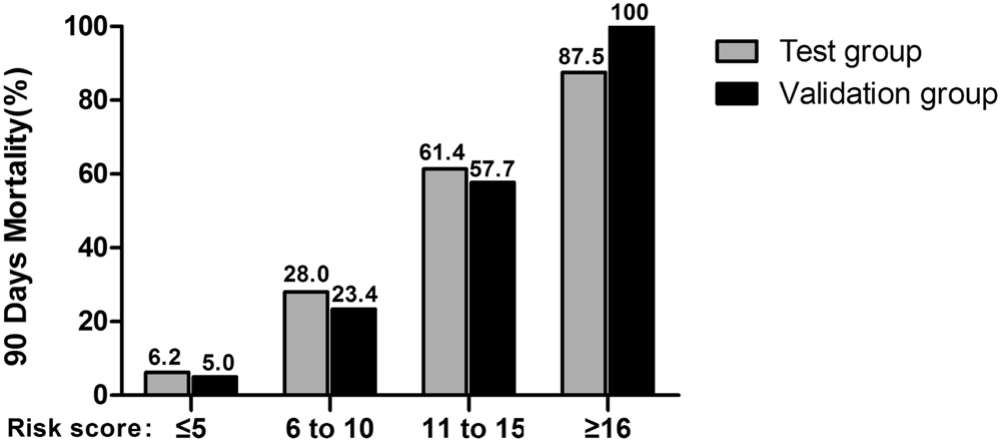
Corresponding 90-day morality for different level of new score between test group and validation group.

The further risk stratification forewarning models were established according to the above 90 days-prognosis scoring system. The test group and validation group were divided into two risk stratification respectively according to the cut-off point: < 5 (low-risk patients), ≥5 (high-risk patients). The mortality rates of the test groups were 5.6% in low-risk patients and 38.5% in high-risk patients. The mortality rates of the validation groups were 4.6% in low-risk patients and 37.6% in high-risk patients. There were no significant differences in AKI death rate in 90-day-prognosis between the test group and validation group by CMH χ^2^ test (Cochran mantel haeszel Statistics). Significant difference was found between patients with low risk and high risk (all p < 0.00), and it was further demonstrated that the higher new score result in the higher cumulative AKI and mortality (Fig. 5).Our result showed that the new scoring model had good prediction ability on AKI mortality rate (Table 6).

**Table 6:** The cut-off point of the new scores for predicting cumulative AKI and mortality rates at 90-day after AKI diagnosis.

Cut-off point of the new score (5.0)	Predicting mortality rate (%)		
	test group	validation group	*P* value
<5	5.6	4.6	0.511
≥5	38.5	37.6	0.894
*P* value	0.000	0.000	

### Comparison of new scores, SOFA and ATN-ISI in predicting 90-day mortality after AKI diagnosis

As for the assessment of calibration, Table 7 lists goodness-of-fit measured by the Hosmer-Lemeshow analysis to predict hospitalized AKI mortality risk and the predictive accuracy of the new scores, SOFA and ATN-ISI in the test and validation groups. In predicting the cumulative AKI and mortality rates, as shown in Tables 7 and 8, Figs 2 and 3, Supplementary Figure S1, Supplementary Table S2 and Supplementary Table S3, new score showed a large AUROC (0.833, 0.832, and 0.830) and stable specificity (82%, 85%, and 82%) in test group, validation group and the second validating dataset, respectively, suggesting that our model showed good stability and repeatability in different datasets. To assess the values of selected cutoff points for predicting AKI mortality, the sensitivity, specificity and positive predictive value were determined (Table 8, Supplementary Table S3). Positive predictive values of new score among test group, validation group and the second validating dataset were 87%, 89% and 90%, while SOFA is 91%, 75% and 84%, ATN-ISI is 90%, 67% and 70%, indicated that all three models had good fitting degree of positive predictive value.

**Table 7:** Calibration and discrimination for the scoring methods in predicting 90-day mortality of patients with AKI diagnosis.

	Calibration			Discrimination		
	Goodness- of-fit	df	*p*	AUROC ± SE	95%CI	*p*
Test group						
New scores	2.839	4	0.585	0.833 ± 0.023	0.788–0.879	0.000
SOFA	10.885	8	0.208	0.784 ± 0.027	0.730–0.838	0.000
ATN-ISI	1.288	7	0.989	0.772 ± 0.028	0.716–0.287	0.000
validation group						
New scores	0.631	4	0.960	0.832 ± 0.035	0.764–0.901	0.000
SOFA	14.369	8	0.073	0.723 ± 0.043	0.638–0.807	0.000
ATN-ISI	8.953	8	0.346	0.719 ± 0.045	0.630–0.808	0.000

Abbreviation: AKI, acute kidney injury; df, degree of freedom; AUROC, areas under the receiver operating characteristic curve; SE, standard error; CI, confidence interval; ATN-ISI: acute tubular necrosis-individual severity index; SOFA: sequential organ failure assessment.

**Table 8:** Comparison of new scores, SOFA and ATN-ISI in predicting 90-day mortality after AKI diagnosis according to Youden index.

Predictive Factors	Cutoff Point	Youden Index	Sensitivity (%)	Specificity (%)	positive predictive value (%)
Test group					
New scores	5.0^a^	0.515	69	82	87
SOFA	6.0^a^	0.507	65	85	91
ATN-ISI	0.23^a^	0.478	63	84	90
Validation group					
New scores	5.0^a^	0.536	69	85	89
SOFA	6.0^a^	0.325	63	70	75
ATN-ISI	0.23^a^	0.363	73	64	67

Abbreviation: AKI, acute kidney injury; ATN-ISI: acute tubular necrosis-individual severity index; SOFA: sequential organ failure assessment; ^a^Value giving the best Youden index.

## Discussion

Excitingly, a new prediction scoring model for 90-day mortality of hospitalized patients diagnosed with AKI was established in our study. It could help clinicians to accurately predict the prognosis of AKI patients, improve quality of life and reduce mortality of AKI patients.

Even with 35 different description of AKI listed in papers worldwide, we lacked a specific and single definition. AKI classification diagnostic criterion is also constantly updating and developing along with the change of the definition. The KDIGO put forward the new classification diagnostic criteria in 2012 on the basis of the improvements of RIFLE and AKIN^8^. Its diagnostic sensitivity and specificity obtained further ascension, so we chose KDIGO criteria in this research.

In our study, the 90-day mortality rate of AKI inpatients was 11.6%~13.8%. Bellomo R and Palevsky PM *et al*. showed that the mortality rate of critically ill patients with AKI was 40%~70%^23, 24^. Sara Nisula reported that mortality rate of 1141 cases of AKI inpatients was 25.6% and the mortality rate of 90-day-progonsis was 33.7%^25^. Other scholars reported that the mortality rate of AKI patients was 32% in 28 days^26^, 44.7% and 52.5% in 90 days^23, 24^ and 57.5% in 2 years^27^. The reasons of different mortality rates might be related to diagnosis standard, patients selection, the improvement of the treatment level, racial difference, basic disease, complication, cultural difference, individual economy and medical insurance difference. With the development of the treatment level and the emergence of new technology, the cure rate would change as well as the mortality.

The identification of risk factors for poor prognosis of AKI patients is required so that preventive and early diagnosis measures can be taken to reduce patients’ mortality. Our previous publication showed that age, AKI types, hypotension, multi-organ failure and ATN-ISI score and K^+^ concentration were the death related independent risk factors of AKI inpatients^28^. Other researchers reported that AKI classification, hypoalbuminemia, hypovolemia before admitting into ICU, baseline serum creatinine, ICU stay, more than four organs function failure, mechanical ventilation, sepsis and AKI attributable to nephrotoxic agents, and oliguria occurrence at AKI diagnosis, were independent significant prognostic indicators, especially preexisting CKD is the greatest known risk factor for the development of AKI^29–36^. Based on our previous work and parameters mentioned above, we observed the death-related risk parameters in this study. Our present results showed that age, AKI types, hypotension, respiratory failure, central nervous system failure and ATN-ISI score were the death independent risk factors in 90 days of the test patients, which suggested that death related risk factors of AKI patients may be related to diagnosis standard and following-term, patient selection, the improvement of the treatment level *et al*.

Also, establishment of forewarning model, identification of patients at high risk and early intervention are the keys to successful rescue and low mortality in AKI patients. The representative AKI prognosis scoring systems included APACHE II, SAPS II, MPM II, ATN-ISI, SOFA and SHARF *et al*. Most study index of the above scoring systems were derived from ICU, which may not suit for all hospitalized AKI patients. Moreover, in China, there has been no large-scale multi-center study and the scope of application was limited. Recently AKI morbidity model after cardiovascular surgery was published, but still has limited scope of application in other types of AKI inpatients^37^. In short, there were few studies on the forewarning model of the prognosis for Chinese AKI inpatients according to KDIGO criteria.

In this study, we focused on the short-term death risk assessment of hospitalized AKI patients with simple size of 1169 patients according to the latest KDIGO criterion. The forewarning model of 90 days after hospitalized patients with AKI diagnosis was built. The mortality rate of total score <5 was 5.6% while the mortality rate of total score ≥5 was 38.5%. The risk stratification forewarning model had good predictive value as observed through validation group. We found that the new scoring model, SOFA and ATN-ISI are all effective in predicting short-term mortality. Moreover, the new scoring model showed a large AUROC and stable specificity in the test group, the validation group and the second validating dataset, respectively, suggesting that our model showed good stability and repeatability in different datasets and may be developed into a potential tool for predicting short-term mortality of hospitalized AKI patients.

This new scoring model can provide some benefits for AKI patients with high risks, such as improvement of the accuracy of nursing grades; early consultation for hospital multi-disciplinary team; timely detection and correction of reversible risk factors. Our medical staff should continue to track and follow up high risk patients after discharge. This new model will help to improve life quality of AKI patients, and reduce medical disputes.

Our study still has some limitations. First, this was a retrospective study which limits the generalization of its findings; Second, it was a single-province multi-center study which may not be directly applicable to other patient populations; Third, we did not do further study on whether the change of new score has a correlation with the long-term prognosis. Therefore, multi-center prospective trials are still necessary to evaluate the accuracy of the new score in predicting 90-day mortality and long-term mortality of AKI patients.

In summary, we established a new prediction scoring model to predict mortality for hospitalized patients with AKI. The predictive mortality rate was close to the actual mortality rate. This prediction scoring model provides a portable tool for clinicians to identify high risk patients early and accurately predict the prognosis of AKI patients to reduce mortality of AKI patients.

## Electronic supplementary material

Supplementary materials
