# Supplementary material for: A new scoring model for the prediction of mortality in patients with acute kidney injury
Source: Sci Rep. 2017 Aug 11;7:7862. doi: 10.1038/s41598-017-08440-w (PMC5554175; doi:10.1038/s41598-017-08440-w)

**A** **new scoring model for the prediction of mortality in patients with acute kidney injury**

Min Luo, Yuan Yang, Jun Xu, Wei Cheng, Xu-Wei Li, Mi-Mi Tang , Hong Liu, Fu-You Liu, Shao-Bin Duan

**Supplementary materials**

**Supplementary Table S1.** Basic data of new dataset from another three hospitals.

**Supplementary Table S2.** Calibration and discrimination for the scoring methods in predicting 90-day mortality of patients with AKI diagnosis in the second validating dataset

**Supplementary Table S3.** Comparison of new scores, SOFA and ATN-ISI in predicting 90-day mortality after AKI diagnosis according to Youden index in the second validating dataset

**Supplementary Figure S1.** Comparison of areas under the receiver operating characteristic curve among new scores, SOFA and ATN-ISI in the second validating dataset

**Supplementary Table S1.** Basic data of new dataset from another three hospitals.

| parameter | New dataset (n=409) |
| --- | --- |
| Age (years, %) |  |
| 15～39 | 95 (23.2%) |
| 40～64 | 181 (44.3%) |
| ≥65 | 133 (32.5%) |
| Gender (male, %) | 252 (61.6%) |
| Baseline Scr (μmol/L) | 88.63 ± 25.3 |
| Baseline eGFR (ml/min/1.73m²) | 78.07 ± 10.1 |
| AKI types |  |
| CA-AKI | 333 (81.4%) |
| HA- AKI | 76 (18.6%) |
| Causes of AKI |  |
| hypovolemia | 121 (29.6%) |
| cardiorenal syndrome | 49 (12%) |
| hepatorenal syndrome | 16 (3.9%) |
| sepsis | 15 (3.7%) |
| organic kidney disease | 82 (20.0%) |
| acute tubular necrosis | 34 (8.3%) |
| post-renal obstruction | 44 (10.8%) |
| multi-factorial | 48 (11.7%) |
| Proteinuria | 242 (59.2%) |
| Hematuresis | 280 (68.5%) |
| Oliguria/anuria (%) | 352 (86.1%) |
| CKD(%) (eGFR<60 ml/min/1.73m²) | 103 (25.2%) |
| Diabetes mellitus (%) | 71 (17.4%) |
| Hypertension (%) | 109 (26.7%) |
| Mechanical ventilation (%) | 73 (17.8%) |
| Hypotension (%) | 86 (21.0%) |
| Organ failure (%) |  |
| heart failure | 86 (21%) |
| hepatic failure | 95 (23.2%) |
| respiratory failure | 70 (17.1%) |
| gastrointestinal failure | 34 (8.3%) |
| central nervous system failure | 69 (16.9%) |
| Hemoglobin < 90g/L (%) | 188 (46%) |
| Hypoalbuminemia (%) | 211 (52%) |
| Scr peak value (μmol/L) | 500.2 ± 367.1 |
| hospital stay (days) | 14.89 ±11.1 |
| Renal replacement therapy (%) | 156 (38.1%) |
| Death in 90days after AKI | 102 (24.9%) |

Abbreviation: Scr: serum creatinine; eGFR: estimated glomerular filtration rate; CA-AKI: Community-acquired AKI; HA-AKI: Hospital-acquired AKI; CKD: chronic kidney disease;

**Supplementary Table S2.** Calibration and discrimination for the scoring methods in predicting 90-day mortality of patients with AKI diagnosis in the second validating dataset

| new | Calibration | | | Discrimination | | |
| --- | --- | --- | --- | --- | --- | --- |
| dataset | Goodness- of-fit | df | p | AUROC±SE | 95%CI | p |
|  |  |  |  |  |  |  |
| New scores | 2.224 | 5 | 0.661 | 0.830±0.025 | 0.783-0.881 | 0.000 |
| SOFA | 10.605 | 8 | 0.225 | 0.732±0.029 | 0.675-0.789 | 0.000 |
| ATN-ISI | 10.290 | 8 | 0.245 | 0.818±0.025 | 0.770-0.866 | 0.000 |

Abbreviation: AKI, acute kidney injury; df, degree of freedom; AUROC, areas under the receiver operating characteristic curve; SE, standard error; CI, confidence interval; ATN-ISI: acute tubular necrosis-individual severity index; SOFA: sequential organ failure assessment.

**Supplementary Table S3.** Comparison of new scores, SOFA and ATN-ISI in predicting 90-day mortality after AKI diagnosis according to Youden index in the second validating dataset

| Predictive Factors in New dataset | Cutoff Point | Youden Index | Sensitivity  (%) | Specificity  (%) | positive predictive value  (%) |
| --- | --- | --- | --- | --- | --- |
| New scores | 5.0^a^ | 0.546 | 73 | 82 | 90 |
| SOFA | 6.0^a^ | 0.416 | 89 | 52 | 84 |
| ATN-ISI | 0.23^a^ | 0.295 | 57 | 73 | 70 |

Abbreviation: AKI, acute kidney injury; ATN-ISI: acute tubular necrosis-individual severity index; SOFA: sequential organ failure assessment; ^a^: Value giving the best Youden index.

**Supplementary Figure S1.** Comparison of areas under the receiver operating characteristic curve among new scores, SOFA and ATN-ISI in the second validating dataset


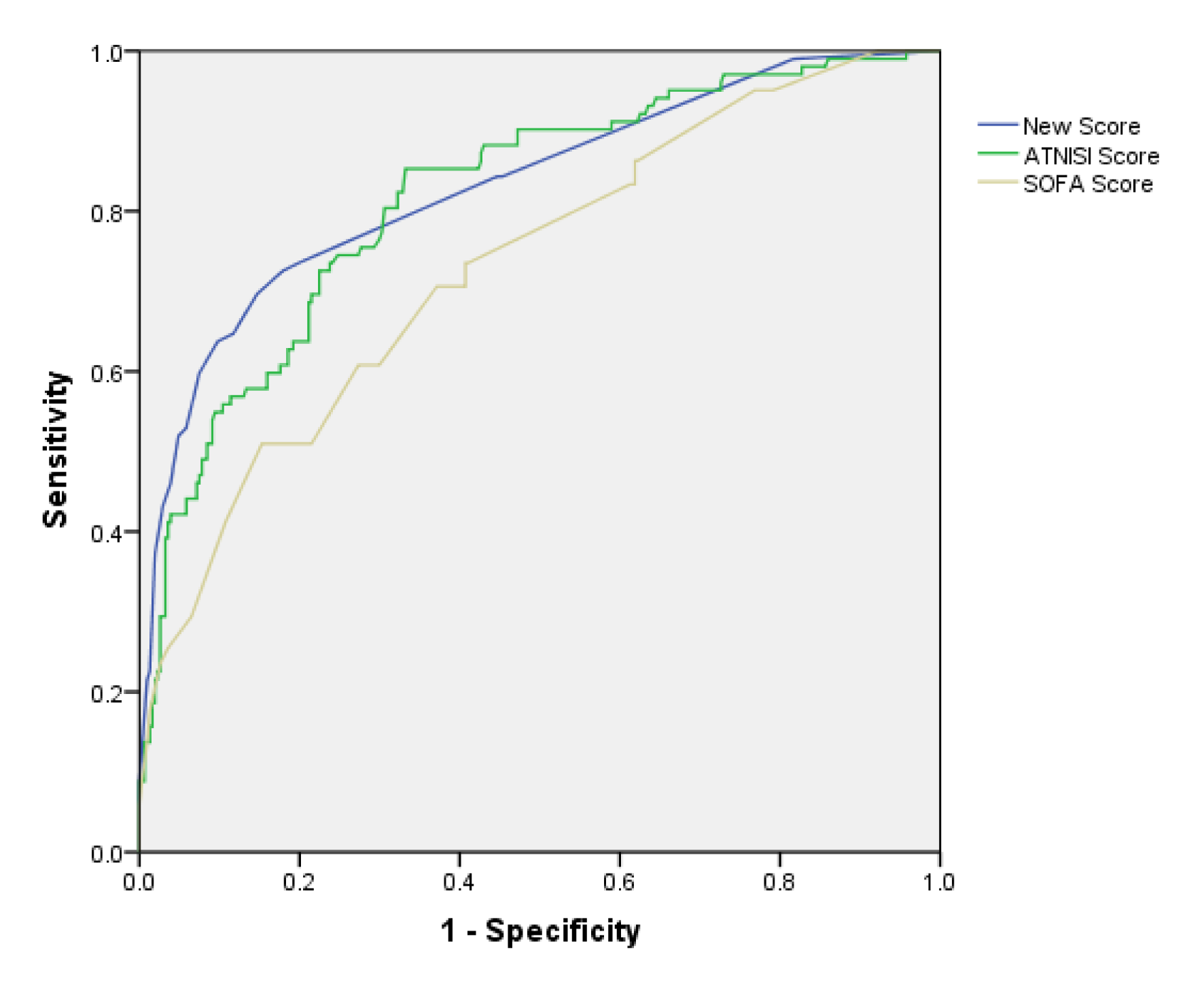

Supplement: Supplementary file 1 — Supplementary materials [file 41598_2017_8440_MOESM1_ESM.doc]
